# Case Report: Subgaleal drainage removal results in a fatal complication after burr-hole evacuation of chronic subdural hematoma

**DOI:** 10.3389/fsurg.2025.1533629

**Published:** 2025-03-19

**Authors:** Lydia Karamani, Donjetë Januzi, Niklas Eckard, Christian Senft, Peter Baumgarten

**Affiliations:** ^1^ Department of Neurosurgery, Jena University Hospital, Friedrich-Schiller University, Jena, Germany; ^2^ Department of Neuroradiology, Jena University Hospital, Friedrich-Schiller University, Jena, Germany

**Keywords:** subgaleal drainage, subdural drainage, chronic subdural hematoma, drainage techniques, Duret hemorrhage

## Abstract

Placement of a drain in subgaleal space in the management of chronic subdural hematomas is a common technique. Subgaleal drains are considered a safe, effective and minimally invasive technique with low-complication rate. In this report, we present a rare but tragic adverse complication following the removal of a subgaleal drainage in a patient who had undergone an evacuation of a subdural bleeding. Although existing data indicates that the risk of cortical surface damage during drain removal is minimal, one must be aware of rare complications such as inadvertent injury to subcutaneous vascular structures culminating in an acute subdural hematoma.

## Introduction

A subdural hematoma is one of the most common diseases operatively treated in neurosurgery. Typically, it results due to mild head trauma in elderly patients. Different operative procedures have been proposed over the years, with the most usual being the burr-hole evacuation. Other operative techniques for evacuating the hematoma involve twist-drill and craniotomy (1). Apart from the difference approaches, various techniques for operating on subdural hematomas have been described. Some studies have reported better postoperative results and a lower recurrence rate with irrigation (2). There is also interest in the type of irrigation used (artificial cerebrospinal fluid, normal saline, half saline) (3), whereas other studies have found no long—term efficacy of irrigation (4). The effectiveness of subdural hematoma surgery can be assessed based on various factors, such as the indication for reoperation, the patient's clinical condition, and mortality. The FINISH trial compared irrigation with non-irrigation in subdural hematoma surgery and found a lower reoperation rate in the irrigation group, as well as no difference in the patient's functional outcome or mortality. Therefore, the FINISH trial suggests the use of subdural irrigation (5). Furthermore, embolisation of the middle meningeal artery, when used in conjuction with conventional treatment, provides a safe and effective treatment option (6). At our institution, subdural hematomas are typically treated with burr-hole trepanation, irrigation of the subdural space with body-temperature normal saline, and insertion of a drain (either subgaleal or subdural). In all cases, the drain used is passive and non-vacuum. Intraoperative audiovisual material of the presented case is not available, and information about intraoperative conditions has been obtained from the surgical report. A burr-hole was drilled at the point of maximum hematoma expansion, the dura mater was incised, and the hematoma was evacuated using a Nelaton catheter and 500 ml–1 L of normal saline, irrigating the subdural space in all directions until clear fluid flowed back.

It is commonly assumed that the placement of a drain reduces recurrence and leads to a better post-operative outcome (7). There are two types of drains after burr-hole evacuation. The standard insertion of a subdural drainage has been used routinely, as it is associated with a better surgical outcome and a lower recurrence (8). The new and innovative technique is the subperiosteal and/or subgaleal drainage. It is proposed as an effective and safe technique with less parenchymal brain tissue injuries (9). Moreover, some studies indicate that the subgaleal drainage is an advantageous method due to the lower rate of recurrence, surgical infections and misplacement (10, 11). Metanalytic studies show a significantly lower rate of recurrence, postoperative bleedings and brain injury (1). Furthermore, patients who obtain subgaleal drainage tend to have fewer epileptic seizures. An explanation for this might be the fact that the drain tube has no direct contact to the parenchyma or the membranes forming on the surface of the subdural hematoma (12). Consequently, the insertion of subgaleal drainage is favored lately by many neurosurgeons. In our neurosurgical center is likewise a well-established technique by some surgeons. However, it remains senior surgeons' choice where the drain is placed.

## Case presentation

An 82-year old man was brought to our emergency department due to the sudden onset of incomplete expressive aphasia. Additionally, the patient had been experiencing a mild weakness in his right leg over the past 2 days. The weakness primarily affected the proximal muscles, with a strength grade of 3/5. The patient's medical history, obtained from a relative due to the challenging communication, revealed chronic arterial hypertension. There was no history of anticoagulation therapy or head trauma.

Upon arrival, a CT scan was conducted to evaluate any intracranial pathology explanatory of the symptoms. Given the absence of a trauma history, the working diagnosis at this stage was an ischemic stroke.

**Figure 1:**
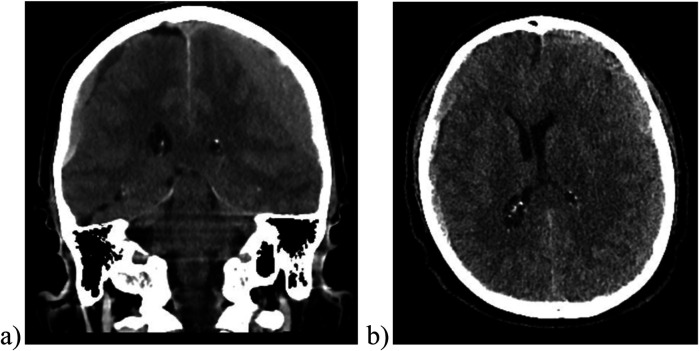
Cranial CT scan images, including coronal **(a)** and axial **(b)** views, reveal a bilateral subdural hematoma. Right-sided hematoma measures approximately 0.8 cm, while the left-sided hematoma about 2 cm, resulting in mass effect and midline shift to the right of approximately 0.9 cm.

The native CT scan revealed an extensive isodense subdural fluid collection that was bilateral, with the left side being predominantly affected and reaching a maximum thickness of 2 cm. On the right side, it extended to a maximum thickness of 0.8 cm. The pressure on the left hemisphere resulted in a noticeable midline shift to the right measuring 0.9 cm (Figure 1). No indications of skull fracture or other intracranial disorder were observed. An ischemic event was further ruled out by performing a contrast enhanced CT scan.

Based on the radiological and clinical findings, an emergent operative treatment was deemed necessary. Consequently, on the same day, we proceeded with the evacuation of the left sided subdural hematoma. The operation was performed through a left frontal burr-hole, followed by the placement of a subgaleal drainage.

**Figure 2:**
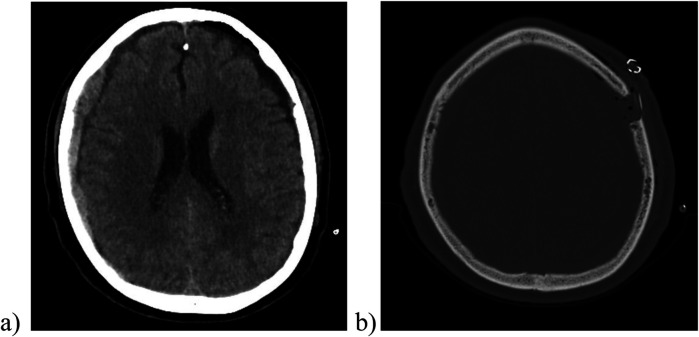
The postoperative cranial CT scan (image **a**, soft tissue window) showed a significant reduction in the size of the left-sided hematoma and the associated midline shift. In the image **(b)** (bone window) one can observe the placement of the subperiosteal/subgaleal drainage (no contact to the underlying brain tissue).

On the first postoperative day, a cranial CT imaging was conducted to evaluate the operating result and to exclude any changes of the right-sided hematoma. This CT scan revealed a notable regression of the left subdural hematoma and, consequently, an almost complete regression of the prior midline shift. The right subdural hematoma remained stable. The postoperative cranial CT scan also depicted the drain in the subcutaneous space (Figure 2).

Following the operation, the patient's clinical condition notably improved. There was a resolution of the speech disorder and the patient could speak fluently, albeit at a slower pace. The right-sided hemiparesis was completely alleviated. Consequently, 48 h post-surgery, we decided to remove the drain.

**Figure 3:**
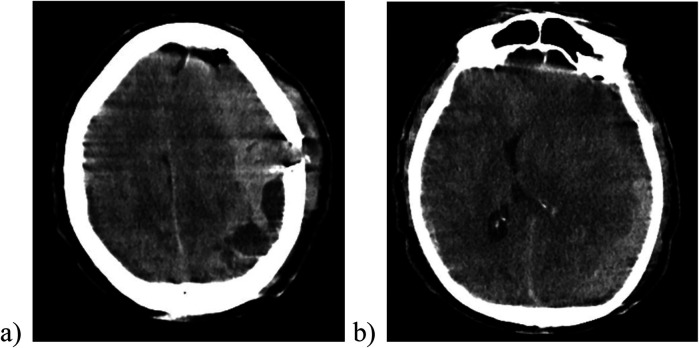
The cranial CT scan after removal of the subgaleal drainage revealing a massive sudden re-bleeding on the left side with midline shift to the right (image **a**), compression of the brain parenchyma and the lateral ventricle (image **b**).

However, shortly afterwards, the patient's clinical condition deteriorated rapidly. The patient was found in an altered mental status, decreased consciousness, and anisocoria. The left pupil was fixed and dilated, unresponsive to light and the Glasgow Coma Score (GCS) dropped to 6/15. An emergency cranial CT scan revealed an acute subdural hematoma on the left hemisphere, measuring up to 32 mm in thickness and exerting a severe mass effect, with a midline shift to the right of about 21 mm, leading to uncal herniation (Figure 3).

**Figure 4:**
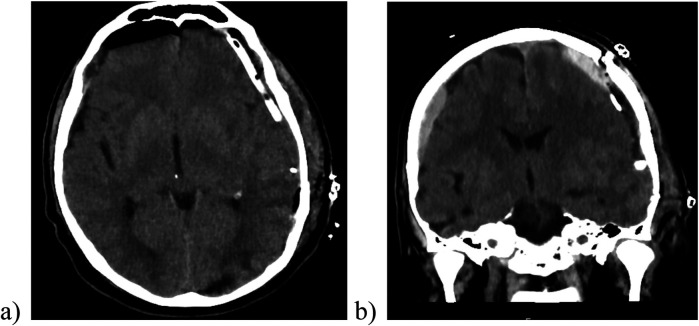
The postoperative cranial CT scan demonstrating a sufficient evacuation of hematoma, the placement of drain (image **a**) and ICP transducer in the subdural space (image **b**).

The urgent evacuation of the left sided hematoma was indicated. The patient was brought immediately to the operating room. During the surgical intervention, a left frontal-to-parietal craniotomy with application of two subdural drainages and a subdural intracranial pressure (ICP) transducer was performed (Figure 4).

Prior to anesthesia induction, the patient experienced four episodes of generalized epileptic seizures, which were successfully halted after administration of anticonvulsant medication.

The postoperative course was complicated with an extended stay in the Intensive Care Unit (ICU). Attempts to extubate the patient failed due to the compromised neurological state, characterized with the absence of protective reflexes and diminished alertness.

An antibiotic treatment was begun on the 7th postoperative day due to pneumonia.

**Figure 5:**
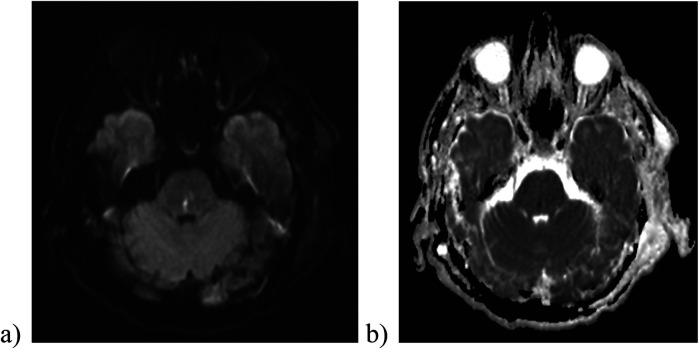
Diffusion-weighted imaging (b1000) **(a)** and ADC image **(b)** reveal the presence of a Duret hemorrhage in the pons.

Early postoperative CT imaging showed a reduction in size of the left-sided hematoma, but also indicated signs of subacute cerebral infarction stemming from the herniation in the left occipital and temporal lobe. Subsequent brain MRI scans confirmed the presence of areas of cerebral ischemia and identified a Duret hemorrhage in pons, a secondary brainstem hemorrhage resulting from descending transtentorial herniation (Figure 5). The occurrence of a Duret hemorrhage is typically associated with an unfavorable prognosis and adverse outcome (13).

An extensive coagulopathy screening was conducted, yielding unremarkable results. The multiplate platelet function analysis did not reveal any abnormalities, and coagulation factors remained within normal range. Subsequent regular screening of hemostatic parameters indicated no signs of coagulopathy.

The patient's health continued to deteriorate in subsequent weeks, and despite intensive medical care, there was no improvement of the neurological status. Regrettably, the patient passed away in the ICU 20 days following the surgical intervention.

## Discussion

The case we have presented highlights a severe and, ultimately fatal, complication following the removal of a subgaleal drainage. Our leading hypothesis regarding the cause of the acute subdural bleeding is an extracranial vascular injury in the vicinity during the drain removal, with blood re-entering the subdural space through the burr-hole in contrast to more common reasons as published before (14). Another hypothesis that could explain the causality of the acute hemorrhage is mechanical tamponade caused by the applied drain, followed by de-tamponade after drain removal. An intraoperative injury to the brain parenchyma can be highly unlikely, as no complications have been reported by the surgical team.

While it might be assumed that having the drainage outside the skull would reduce the risk of intracranial bleeding during removal, the presence of an open burr-hole could, conversely, increase the risk of blood entering the subdural space. It remains unclear whether covering the burr-hole could prevent the accumulation of blood in the subdural space, as it might still interfere with the purpose of drain insertion.

In a meta-analysis comparing subdural with subgaleal drainage, lower morbidity was observed among patients receiving subgaleal drainages. The incidence of drain misplacement, infection or parenchymal injury was significantly higher in patients with subdural drainage (10).

Another randomized trial that investigated the choice of drainage in patients with subdural hematoma receiving either antiplatelet therapy or anticoagulants reported a higher recurrence rate after subdural drainage placement compared to subperiosteal ones. Although this difference did not reach statistical significance, it may be attributed to the fact that the placement of subgaleal drainage involves less subdural manipulation, thus reducing the risk of vessel injuring (15). A retrospective study comparing the two drainage techniques, subgaleal vs. subdural, revealed no statistically significant difference in recurrence rates among patients with chronic subdural hematoma. However, in subacute hematomas, it was found that subgaleal drainage is associated with a higher recurrence rate (16).

The choice of drainage in patients with subdural hematoma is ultimately the decision of the neurosurgeon, and there is no consensus on the preferable type of drainage. In a study by Soleman et al., international preferences of drainage types were investigated. The majority of neurosurgeons (80%) preferred to insert a drainage, 16% did so occasionally and 4% refrained from inserting one. Of those who opted out for placing a drainage, 50% preferred a subdural and 27% a subgaleal one. Interestingly, in 23% of cases, subdural drainage was the initial preference and only if not feasible, subgaleal placement followed. It is noteworthy that older neurosurgeons with over 10 years of experience tended to place subdural drainage more frequently, while younger colleagues preferred subgaleal drainages (17).

These results may reveal a recent trend that has shifted the perspective on the choice of drainage in patients with subdural hematomas.

## Conclusion

A fatal complication as shown underscores the importance of carefully justifying and deliberating the decision of type of drain.

## Data Availability

The raw data supporting the conclusions of this article will be made available by the authors, without undue reservation.
